# Neural bases of enhanced attentional control: Lessons from action video game players

**DOI:** 10.1002/brb3.1019

**Published:** 2018-06-19

**Authors:** Julia Föcker, Daniel Cole, Anton L. Beer, Daphne Bavelier

**Affiliations:** ^1^ Faculty of Psychology and Educational Sciences Ludwig‐Maximilians‐University Munich Germany; ^2^ University of Rochester Rochester New York; ^3^ University of Regensburg Regensburg Germany; ^4^ University of Geneva Geneva Switzerland

**Keywords:** action video games, attentional control, bottom‐up attention, frontoparietal brain networks, neural plasticity, top‐down attention

## Abstract

**Objectives:**

The ability to resist distraction and focus on‐task‐relevant information while being responsive to changes in the environment is fundamental to goal‐directed behavior. Such attentional control abilities are regulated by a constant interplay between previously characterized bottom‐up and top‐down attentional networks. Here we ask about the neural changes within these two attentional networks that may mediate enhanced attentional control.

**Materials and Methods:**

To address this question, we contrasted action video game players (AVGPs) and nonvideo game players (NVGPs) in a Posner‐cueing paradigm, building on studies documenting enhanced attentional control in AVGPs.

**Results:**

Behavioral results indicated a trend for more efficient target processing in AVGPs, and better suppression in rare catch trials for which responses had to be withheld. During the cue period, AVGPs recruited the top‐down network less than NVGPs, despite showing comparable validity effects, in line with a greater efficiency of that network in AVGPs. During target processing, as previously shown, recruitment of top‐down areas correlated with greater processing difficulties, but only in NVGPs. AVGPs showed no such effect, but rather greater activation across the two networks. In particular, the right temporoparietal junction, middle frontal gyrus, and superior parietal cortex predicted better task performance in catch trials. A functional connectivity analysis revealed enhanced correlated activity in AVGPs compared to NVGPs between parietal and visual areas.

**Conclusions:**

These results point to dynamic functional reconfigurations of top‐down and bottom‐up attentional networks in AVGPs as attentional demands vary. Aspects of this functional reconfiguration that may act as key signatures of high attentional control are discussed.

## 1 INTRODUCTION

Attentional control is crucial to our everyday behavior, allowing us to filter through the vast amount of information we are confronted with all the while remaining aware of possible changes in our environment. Key attentional control mechanisms include focusing on specific locations, times or objects of interest, filtering out noise or distractions as well as allocating attentional resources in a task optimal manner. Attentional control allows for flexible adaptation as task goals or environmental demands shift and is thus a building block of well‐adapted behavior.

The possibility of enhancing attentional control has understandably become a topic of interest given the many benefits such enhancement would attain. Among the interventions hypothesized to potentially benefit from attentional control are various forms of rather complex training regimens, including physical exercise, mind‐brain meditation techniques, playing a musical instrument, working memory training, and playing action‐packed video games. The possibility of enhancing attentional control through these techniques has been explored throughout the life span from children to older adults (*video games*: Bavelier, Green, & Dye, 2010; Green & Bavelier, 2012; *physical exercise*: Voss, Nagamatsu, Liu‐Ambrose, & Kramer, 2011; *working memory training*: Klingberg, 2010; *meditation*: Gard, Hölzel, & Lazar, 2014; Mooneyham, Mrazek, Mrazek, & Schooler, 2016; Slagter, Davidson, & Lutz, 2011; *musical training*: Schellenberg, 2015).

A key point in the present work concerns the neural mechanisms that mediate attentional control enhancements. So far, we know about the networks that support attentional control, in particular the interplay between the bottom‐up guidance of attention and its top‐down control (Asplund, Todd, Snyder, & Marois, 2010; Buschman & Miller, 2007; Corbetta, Patel, & Shulman, 2008; Fox, Corbetta, Snyder, Vincent, & Raichle, 2006; Hopfinger, Buonocore, & Mangun, 2000; Leitão, Thielscher, Tünnerhoff, & Noppeney, 2015; Serences, Yantis, Culberson, & Awh, 2004; Serences et al., 2005; Sylvester, Shulman, Jack, & Corbetta, 2007; Vossel, Geng, & Fink, 2014; Wu et al., 2015). For instance, a common neuroanatomical model of attentional control has been proposed (Corbetta et al., 2008) including (a) a goal‐directed (top‐down) network whose core regions consist of a dorsal fronto‐parietal network including the intraparietal sulcus (IPS), the superior parietal lobule (SPL) and the frontal eye field (FEF) and (b) a more ventral stimulus‐driven (bottom‐up) network consisting of the temporal parietal junction (TPJ), the anterior insula and the medial frontal gyrus (MFG) (Corbetta et al., 2008 for a review; Corbetta & Shulman, 2002 for a review; Giesbrecht, Woldorff, Song, & Mangun, 2003; Hopfinger et al., 2000; Nardo, Santangelo, & Macaluso, 2011). A dominant view is that these two networks mainly interact with each other via the medial frontal gyrus (MFG) (Corbetta et al., 2008; Corbetta & Shulman, 2002; Fox et al., 2006; He et al., 2007; Vossel et al., 2014). A number of studies document that top‐down and bottom‐up interactions also engage parietal areas ‐ including the lateral intraparietal cortex (LIP), the intraparietal sulcus (IPS) (Buschman & Miller, 2007; Gottlieb, 2007; Leitão et al., 2015), the temporal parietal junction (TPJ) (Wu et al., 2015) and frontal areas (Asplund et al., 2010; Serences et al., 2005) – including the inferior frontal junction (IFJ) (Asplund et al., 2010) or even more anterior parts of the ventral network (He et al., 2007).

To date, there is less evidence about how these two networks and associated brain areas may support enhanced attentional control. The TPJ has been suggested to be especially recruited during the reorientation of attention and the suppression of distracting stimuli (see Corbetta et al., 2008 for a review). One hypothesis holds that the TPJ, especially in the right hemisphere, sends an early reorientation signal that “circuit breaks” ongoing top‐down attentional processes in regions of the dorsal attentional network (FEF and MFG). Such a role for the right TPJ has, however, recently been called into question (Geng & Vossel, 2013; Silvetti et al., 2016; Vossel, Geng, & Friston, 2014; Vossel et al., 2014). An alternative view is that the TPJ is more involved in “contextual updating” and adjustments of top‐down expectations as task demands may vary. Interestingly, a recent study has documented different patterns of TPJ recruitment in less versus more easily distracted individuals. Individuals less easily distracted activated the TPJ similarly regardless of the pull of the distractors (e.g., new object, luminance decrement). By contrast, highly distractible individuals showed weak TPJ activation in the context of low distraction and only sustained TPJ activation under high distraction. It has been suggested that those individuals who actively recruited the TPJ across distractor conditions might have a more efficient control system (Kim & Hopfinger, 2010). More recently, the TPJ has been proposed to act as both a filter and a trigger depending on the attentional load of the task. In this view, task‐relevant stimuli would activate the TPJ and act as a trigger to reorient attention toward these dimensions under low load, but under high load, the TPJ would mostly permit efficient filtering of irrelevant information (Wu et al., 2015). This perspective predicts a differential recruitment of the TPJ under low load (activation) or high load (inhibition), which has been documented in a few studies (Anticevic, Repovs, Shulman, & Barch, 2010; Shulman, Astafiev, McAvoy, d'Avossa, & Corbetta, 2007; Shulman et al., 2003; Wu et al., 2015).

To investigate how top‐down and bottom‐up attentional control networks are modified in individuals with enhanced attentional control, we compare their recruitment and interactions in two populations known to differ in their attentional control skills, in particular participants who are regular players of action video games (AVGPs) versus those who do not play any kind of action video games (NVGPs). The body of studies available indicates that AVGPs have enhanced attentional control (see Green & Bavelier, 2012). AVGPs have been shown to possess larger attentional resources and display more flexibility in how they distribute these resources over space, time or to objects, which allows them to adapt to task demands (for reviews see Green & Bavelier, 2012; Spence & Feng, 2010). Eye‐tracking studies provide a convergent view by documenting initial oculomotor capture effects that are similar in AVGPs and NVGPs, but swifter recovery in AVGPS when wrongly cued (Chisholm, Hickey, Theeuwes, & Kingstone, 2010; Chisholm & Kingstone, 2012, 2015). Search studies using manual reaction times, detection rate or eye‐tracking concur to demonstrate that AVGPS outperform NVGPs in detecting targets among distractors with some studies directly documenting fewer attention shifts (saccades) to task‐irrelevant distractors (Castel, Pratt, & Drummond, 2005; Chisholm & Kingstone, 2011, 2014, 2015; Clark, Fleck, & Mitroff, 2011; Hubert‐Wallander, Green, Sugarman, & Bavelier, 2011; Mack, Wiesmann, & Ilg, 2016; but see for another view Heimler, Pavani, Donk, & van Zoest, 2014). Interestingly, recent brain imaging data complement these findings. A recent fMRI study revealed less increase in the frontoparietal, top‐down attention network in AVGPs as compared to NVGPs when the attentional load increased during a visual search task (Bavelier, Achtman, Mani, & Föcker, 2012). A possible interpretation is that attentional networks may be less taxed in AVGPs than in NVGPs as the task becomes more difficult, due to a more automatic allocation of attention. Indeed, reduced brain activation and its relation to higher automatization have also been documented in other studies. For example, reduced activation and reduced functional coupling in frontal brain areas, such as the inferior frontal junction (IFJ) has been observed in individuals with high cognitive flexibility, for instance, during task switching (Armbruster, Ueltzhöffer, Basten, & Fiebach, 2012). Other studies suggest that activation within a brain region tends to decrease with task practice and in turn with improvements in performance (Asaad, Rainer, & Miller, 1998; Beauchamp, Dagher, Aston, & Doyon, 2003; Erickson et al., 2007; Jansma, Ramsey, Slagter, & Kahn, 2001; Landau, Schumacher, Garavan, Druzgal, & D'Esposito, 2004; Milham, Banich, Claus, & Cohen, 2003; Raichle et al., 1994; Schneiders et al., 2012). One plausible interpretation for such effects is that the brain implements the needed computations more efficiently, and therefore requires fewer computational resources to accomplish the same processing, which might lead to a more precise functional circuitry (Garavan, Kelley, Rosen, Rao, & Stein, 2000; Karni, 1995). Of course, as load or difficulty increases, the task becomes more effortful for AVGPs, reducing the group differences that may be seen at intermediate loads or difficulty levels. In addition, both brain imaging and electrophysiological data provide evidence for more efficient filtering of irrelevant information in AVGPs during attentionally demanding tasks (Bavelier et al., 2012; Krishnan, Kang, Sperling, & Srinivasan, 2013; Mishra, Zinni, Bavelier, & Hillyard, 2011; Wu et al., 2012). In these experiments, unattended task‐irrelevant stimulus features were observed to elicit less activation in AVGPs than NVGPs suggesting better filtering of distraction or disruption sources, at least under the high load conditions used in these studies. These functional changes may be accompanied by structural changes with one study reporting gray matter changes in the left dorsolateral prefrontal cortex, a part of the attentional network, in experienced video game players (although no specific game genre was specified) compared to nonvideo game players (Kühn et al., 2014).

**Figure 1:**
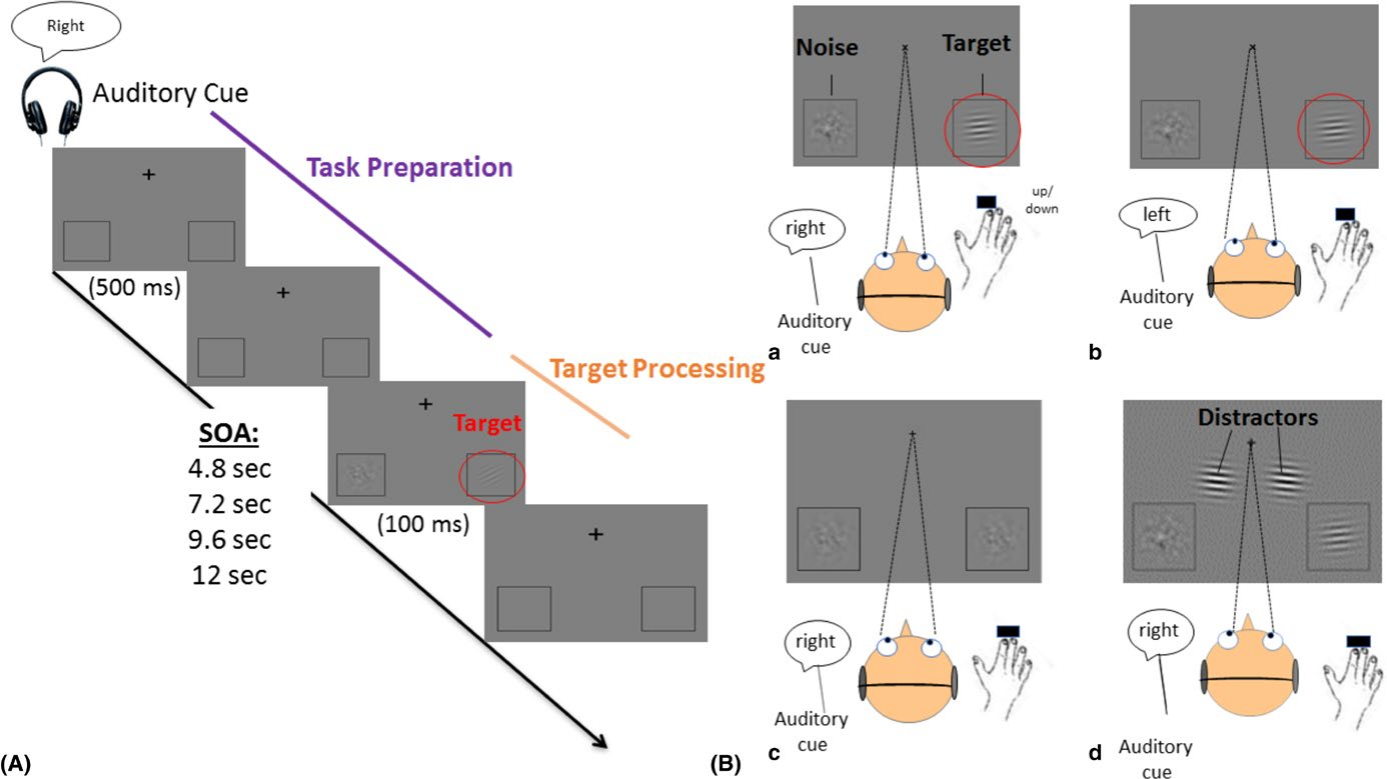
Experimental task. (A) Trial sequence. Each trial started with an auditory cue saying “left” or “right”. After a variable stimulus onset asynchrony (SOA), the visual target was presented either at the cued or uncued location. The participants had to indicate the orientation of the Gabor patch target as fast and as accurately as possible. The presentation of the visual stimuli was followed by a variable intertrial interval. (B) Trial conditions. (a) On standard valid trials (upper left), the Gabor patch was presented at the cued side. (b) On standard invalid trials (upper right), the Gabor patch was presented at the noncued side, requiring a reorientation of attention. (c) On catch trials (lower left), two noise patches were presented in the left and right visual field and participants had to withhold their response. (d) On distractor trials (lower right), high contrast distractors appeared simultaneously with the low‐contrast Gabor and noise patch. Standard valid and standard invalid trials are called target‐present standard trials and distractor valid and distractor invalid trials are called target‐present distractor trials

Better filtering of distractions and lesser recruitment of the frontoparietal network as the load on attentional control initially increases are in line with the enhanced attentional control documented in AVGPs. Yet, these studies fail to properly document how the bottom‐up and top‐down attention networks interact to service enhanced attentional control. To investigate this question, we contrasted AVGPs and NVGPs in an fMRI paradigm known to engage these two attentional networks and designed to highlight how they interact. A cross‐modal cueing paradigm was used whereby on each trial the participant was cued auditorily (voice indicating “left” or “right”) to attend to one of two marked locations in their lower visual fields. Participants were instructed to indicate as fast and as accurately as possible the up or down orientation of a Gabor patch target presented at the same time as a noise patch, with one appearing at the cued location and the other one at the uncued location (see Figure 1). The co‐occurrence of a noise patch prevents identification of the target location via an abrupt onset. In our experiment, the cue was predictable 60% of the time. Thus, by enforcing the maintenance of attention at the predicted target location in a top‐down manner (Posner, 1980), the cue established a period of time during which top‐down attention was engaged as participants prepared for the stimulus to appear.

Notably, on a small percentage of trials, a re‐evaluation of task contingencies had to be performed. First, 20% of the trials were catch trials whereby two noise patches were presented and participants were instructed to withhold response on such trials. Second, on another 20% of the trials, the target appeared at a different location than the cued one (invalid trial), requiring a reallocation of attention over space. Finally, and crossed with validity, on 25% of the target‐present trials, additional, high contrast distractors were presented at the same time as the target and noise patch, near to the two possible target locations (distractor trials). Thus, in distractor trials, participants had to filter out high contrast, salient target‐like distractors (see Figure 1). Standard valid and standard invalid trials are called target‐present standard trials and distractor valid and distractor invalid trials are called target‐present distractor trials.

The frontal eye field (FEF) especially in the right hemisphere, is expected to play a role during the top‐down preparatory activity after the auditory cue has been presented. Indeed, the right FEF shows strong anticipatory activity when participants expect to see an object at a particular location (Corbetta, Kincade, Ollinger, McAvoy, & Shulman, 2000; Kastner, Pinsk, De Weerd, Desimone, & Ungerleider, 1999; Shulman et al., 2009). Yet, the direction of such an effect in participants with enhanced attentional control remains unclear.

On the one hand, enhanced attentional control may lead to better performance and consequently reduced activation in the frontoparietal network, as participants with higher attentional control (in our case, action video game players) can maintain a high level of performance with lesser effort (Armbruster et al., 2012; Bavelier et al., 2012). On the other hand, enhanced attentional control may be mediated by a greater recruitment of the dorsal frontoparietal network, as illustrated by training studies which document increased frontoparietal network recruitment related to increased performance in working memory or attentional control tasks (Brefczynski‐Lewis, Lutz, Schaefer, Levinson, & Davidson, 2007; see Klingberg, 2010 for a review). A distinctive advantage of the paradigm used here is its natural ability at decoupling top‐down, task preparation activation from bottom‐up, stimulus processing activation (see Corbetta & Shulman, 2002). This paradigm allows us to document how the preparatory activity triggered by the auditory cue differs between AVGPs and NAVGPs separately from the attentional processes at play during target processing. During target processing, the TPJ has been shown to be relevant in reorienting attention and ignoring distractors (Corbetta & Shulman, 2002; Corbetta, Patel, & Shulman, 2008; Doricchi, Macci, Silvetti, & Macaluso, 2010; Geng & Vossel, 2013). Thus, we expected to find differences in this region in AVGPs as compared to NAVGPs, with possibly greater TPJ recruitment in AVGPs, mirroring their greater ability at flexibly allocating attention and filtering distractions (Bavelier & Föcker, 2015; Chisholm & Kingstone, 2012; Green & Bavelier, 2012; Krishnan et al., 2013).

## 2 METHODS

### 2.1 Participants

Male participants, 21 action video game players (AVGPs) and 19 nonvideo game players (NVGPs), were initially recruited for this study from the University of Rochester (Rochester, NY, USA). The final sample included 16 AVGPs (mean age 21.1 years; range 18–27 years) and 16 NVGPs (mean age 21.5 years; range 19–26 years). All 32 participants were right‐handed except for one left‐handed NVGP participant; they were all enrolled as students at the University of Rochester, except for one AVGP who graduated from high school but was not a University student.

The final sample was reduced due to the following constraints and exclusion criteria. Two AVGPs datasets were incomplete due to technical problems; an error at recruitment led to the initial misclassification of 2 participants as NVGPs; as these participants qualified as neither AVGPs nor NVGPs, they were excluded from the analyses. In addition, the following exclusion criteria were applied: (a) being an expert in other domains documented to affect cognition (expert music players, athletes or mind‐brain training expert – 1 NVGP excluded for being a music expert); (b) being a high media multitasker as defined by Ophir, Nass, and Wagner (2009) ‐ MMI score >5.9; 3 AVGPs excluded). This 2009 study documented an association between high levels of media multitasking and deficits in cognitive control, in particular in handling distractions. As our interest is in individuals with enhanced attentional control, all so‐defined high media multitaskers were excluded.

Inclusion criteria for AVGPs and NVGPs were based on replies to a videogame usage questionnaire that can be obtained upon request from the Bavelier lab. Those participants who reported playing first or third‐person shooter video games for at least 5 hours per week over the past year were categorized as AVGPs. Those participants who reported playing between 3–5 hours/week of first or third‐person shooter in the past year were also included as AVGPs, if they also reported playing, before the past year, at least 3 hr/week of any of these three game genres: first/third‐person shooters, action‐sports or real‐time strategy video games. Action video games were initially defined as shooter games (first or third person). More recently this criterion has been relaxed as other genres have been documented to produce similar benefits such as action‐sports‐adventure video games and real‐time strategy video games (Bediou et al., 2018; Dale & Green, 2017a,b; Glass, Maddox, & Love, 2013). For this sample, ran between 2011–2012, commonly cited titles included Halo, Call of Duty, Borderlands, Half Life, Counter Strike, Team Fortress 2, Bioshock, Fallout, Killing Floor, or Resident Evil. NVGPs were selected based on reporting no first or third‐person shooter game play in the past year and no more than one hour per week in the year before that. In addition, participants reporting more than 1 hr/week of play in any other video game genre and more than 6 hr/week when adding all game genres usage were excluded from the NVGPs.

All participants had normal or corrected‐to‐normal visual acuity in both eyes as tested by high contrast ETDRS format charts with Sloan optotypes (catalog No. 2104; Precision Vision, La Salle, IL, USA). For those participants who needed corrections (4 participants), MR‐compatible glasses were provided by a trained optometrist. All participants were volunteers that gave written informed consent. The study was approved by the Research Subject Review Board of the University of Rochester, which abides by the Declaration of Helsinki.

### 2.2 Task

The attentional control paradigm was adopted from Sylvester et al. (2007). Participants viewed a screen with a central rotating cross and two locator squares positioned in the lower visual field (4.2 degrees of visual angle from the center; see Figure 1). Participants were asked to fixate on the central cross throughout the trials. At the beginning of each trial, a sound cue (female voice of 500 ms duration saying “left” or “right”) instructed participants to attend to one of the two squares. The sound cue indicated in 60% of the trials the location of an upcoming visual target. After a variable stimulus onset asynchrony (SOA) of either 4.8 s, 7.2 s, 9.6 s, or 12.0 s, two visual stimuli (duration 100 ms) appeared in each of the squares. On 80% of the trials, one of the visual stimuli consisted of a Gabor patch (target), whereas the other consisted of a noise patch (see also Table 1).

**Table 1:** Conditions

Total number of trials: *N *= 160 trial				
Standard trials *N *= 96 trials		Distractor trials *N *= 32 trials		Catch trials *N *= 32 trials
**Validly cued** Gabor Patches (cue indicates the correct location of the upcoming Gabor Patch)	**Invalidly cued** Gabor Patches (cue indicates the wrong location of the upcoming Gabor Patch)	**Validly cued** Gabor Patches **with Distractors**	**Invalidly cued** Gabor Patches **with Distractors**	**Two Noise Patches**
*N *= 72 trials	*N *= 24 trials	*N *= 24 trials	*N *= 8 trials	*N *= 32 trials

*Note*

The table lists the descriptions of the different conditions in the current experiment and the corresponding number of trials (N).

The Gabor patch showed a luminance modulated‐oriented grating with a spatial frequency of 3.5 cycles per degree, whereas the noise patch showed pixelated noise of the same mean luminance. Both the Gabor and noise patch were modulated by a Gaussian with a sigma of 0.4 degrees of visual angle and presented at 4.2 degrees of visual angle from the fixation. The Gabor grating was oriented either 85° (bottom‐left to top‐right) or −85° (top‐left to bottom‐right). Twenty percent of the trials consisted of catch trials during which only two noise patches were presented (target‐absent trials). On 25% of the target‐present trials, the target Gabor and noise patch (inside the squares) were flanked by two high contrast Gabor patches appearing at an eccentricity of 1.6 degrees between the fixation cross and the stimulus squares. These additional patches served as distractors. Participants were asked to indicate the orientation of the Gabor patch in the locator square by pressing one of two buttons with their index or middle finger as fast and as accurately as possible. They were asked to withhold their response during catch trials. The next trial started after a variable intertrial interval (ranging from 2.4 s to 25.2 s) relative to the onset of visual stimuli.

In order to guarantee fixation, the cross in the middle of the screen was rotating and occasionally one arm of the cross was missing (65 missing arms in total, block 1: *n *= 7; block 2: *n *= 10; block 3: *n *= 8; block 4: *n *= 9; block 5: *n *= 5; block 6: *n *= 9; block 7: *n *= 9; block 8: *n *= 8). Participants were asked to count the number of times an arm was missing in addition to performing the main Gabor discrimination task.

The study was comprised of two fMRI sessions each lasting about 1.5 h and conducted on a separate day. In total, 160 trials were presented per session (Table 1). Among these trials, 96 (60%) were standard trials, 32 (20%) were distractor trials and the remaining 32 (20%) trials were catch trials (only two noise patches). Across all trials, 60% were validly cued and 20% were invalidly cued. Among standard trials, 72 were validly cued and 24 were invalidly cued. Among distractor trials, 24 were validly cued and 8 were invalidly cued.

The total set of trials was distributed across eight blocks (runs) consisting of 20 trials each. The order (and intertrial interval) of trials was pseudorandom and optimized for an event‐related fMRI acquisition by the Optseq program developed by Greve (http://surfer.nmr.mgh.harvard.edu/optseq ).

Prior to the first session (separate day), all participants were trained on the task outside the scanner room. During each of the fMRI sessions, the same task was repeated. Yet, the contrast of the Gabor and noise patches was adjusted to the 79% threshold of each participant at the start of the first fMRI session, but fixed to a Michelson contrast of 0.25 in the second fMRI session. The adjustment aimed to equate for contrast sensitivity as previous research showed that video gamers may have lower contrast thresholds (Li, Polat, Scalzo, & Bavelier, 2010). Accordingly, contrast thresholds were assessed at the beginning of the first session inside the MR scanner using a 3‐to‐1 staircase procedure (initial Michelson contrast value of 1.0) with a Gabor patch and task similar to that used in the main task. This procedure yielded a contrast threshold at about 79% correct discriminations (Levitt, 1971). The mean contrast thresholds were 0.17 (SE = 0.03) for AVGPs and 0.22 (SE = 0.04) for NVGPs. Although in the expected direction, the two groups did not differ (*p *=* *0.283). As no significant differences across groups and sessions were observed with regard to the main fMRI results, both fMRI sessions were analyzed together.

### 2.3 Stimulus presentation

Stimuli were presented with the Psychophysics Toolbox (Brainard, 1997; Pelli, 1997) based on Matlab (MathWorks, Natick, MA, USA) on a Power Macintosh G4 computer (Apple, Cupertino, CA, USA). Auditory stimuli were generated by Audacity and were presented using pneumatic headphones in the scanner (Resonance Technology Inc., CA). Visual stimuli were projected onto a screen located in the rear of the magnet bore using a Titan model sx+ 3D projector (Digital Projection, Inc., GA, USA). The screen was visible to the participants through a small mirror mounted above the eyes at an angle of 45°. The viewing distance was usually 80 cm. However, due to temporary modifications in the projection system of the scanner room, seven participants (5 AVGPs and 2 NVGPs) were scanned with a viewing distance of 110 cm. This modification slightly changed the eccentricities of the locator squares (from 4.2 to 3.9 degrees of visual angle) and of the distractors (from 1.6 to 1.5 degrees).

### 2.4 MRI acquisition

Magnetic resonance images were acquired with a Siemens Trio 3T MRI equipped with an eight‐channel head coil. During each of the two sessions, eight fMRI runs (T2*‐weighted) were acquired with a gradient‐echo (GE) sequence with echo‐planar read‐out (EPI) along 36 interleaved axial slices covering the entire brain (TR = 2.4 s, TE = 30 ms, flip angle = 90°, slice thickness = 4 mm, in‐plane resolutio*n *= 4 × 4 mm², field of view = 256 × 256 mm²). Each run contained between 132 and 150 volumes (depending on the trial sequence, see above). Trial presentation started after the fifth volume to assure that magnetization reached equilibrium. Additionally, three‐dimensional T1‐weighted structural images were acquired in each session by a magnetization‐prepared, rapid‐acquisition gradient‐echo (MPRAGE) sequence along 192 sagittal slices (TR = 2530 ms, TE = 3.44 ms, flip angle = 7°, slice thickness = 1 mm, in‐plane resolutio*n *= 1 × 1 mm², field of view = 256 × 256 mm²).

### 2.5 Image preprocessing

The fMRI analysis was performed using FEAT (fMRI Expert Analysis Tool), which is part of the FSL software package version 6.0.0 (Smith et al., 2004; Woolrich, Behrens, Beckmann, Jenkinson, & Smith, 2004). fMRI preprocessing followed procedures as reported in Bavelier et al. (2012). Motion correction was applied to each run using MCFLIRT which corrects with respect to a volume of reference (the middle volume in our case) (Jenkinson, Bannister, Brady, & Smith, 2002). Volumes with head movements greater than 4 mm were detected in three runs (0.01% across all, <14.6% per participant). These outlier volumes were excluded from the analysis by nuisance predictors in the general linear model (GLM). Further preprocessing steps included: slice time correction (interleaved); nonbrain removal using BET (Smith, 2002); spatial smoothing using an isotropic 3D Gaussian kernel (full‐width‐half‐maximum = 5 mm); grand mean‐based intensity normalization; and high‐pass temporal filtering with a 50s cut‐off. All images were linearly registered to a standard brain (MNI‐152 template) using FLIRT (Jenkinson & Smith, 2001).

Following preprocessing, three different types of analyses were carried out: First, whole‐brain analyses aimed to characterize overall group overlap and differences in BOLD activation. Second, a region of interest (ROI) analysis was performed in order to identify brain areas whose activity shows a strong relationship with behavioral outcome. Third, a psychophysiological interaction (PPI) analysis aimed to reveal the functional connectivity of the brain. The GLM approach was performed using the FILM (FMRIB's Improvised Linear Model) tool for a fixed effects single‐subject analysis, followed by a FLAME (FMRIB's Local Analysis of Mixed Effects) (Beckmann, Jenkinson, & Smith, 2003; Woolrich et al., 2004) group analysis. For each of the three analyses, data from both sessions (first and second) were first run separately in each subject and then combined per subject by a fixed effects analysis (using FLAME) as a control analysis revealed no relevant differences across sessions. Coefficient maps were computed for each contrast and subject. All reported analyses are based on all trials (correct and incorrect). Some conditions such as for example catch trials had too few trials to only focus on correct trials.

#### 2.5.1 Whole‐brain group analysis

We used a GLM that modelled the following events as regressors: cue, SOA, target (Gabor present) and catch (only noise patches). Note that target‐present standard trials (only Gabor and noise without distractor) and target‐present distractor trials (Gabor and noise and two distractors) were modelled by one regressor. For within‐group analyses, statistical parametric maps of z‐values (Gaussianised T/F) of the mixed effects model using FLAME were thresholded using a voxel level at *p *< 0.01 (*z *> 2.3) and a cluster level at *p *< 0.05, corrected for multiple comparisons (Worsley, 2001). Separate statistical maps per group were overlaid on the MNI template of FSL. In addition, a between‐group mixed effects analysis using FLAME was performed for each contrast. This analysis compared coefficient maps between AVGPs and NVGPs and served to identify group differences. Again, statistical images of z‐values (Gaussianised T/F) were thresholded using clusters determined by a voxel level at *p *< 0.01 (*z *> 2.3) and a cluster level at *p *< 0.05, corrected for multiple comparisons (Worsley, 2001).

#### 2.5.2 Regions of interest definition

The goal of this analysis was to characterize for our sample of participants the exact locations of the frontoparietal areas documented to mediate attentional control (Corbetta & Shulman, 2002). To this end, a GLM with regressors, cue, SOA and visual stimuli was used and ROIs were defined based on the group contrast AVGP greater than NVGP in the condition all visual stimuli vs. baseline. Note that target‐present trials (standard and distractor ones) as well as catch events (only noise patches) were modelled by the one regressor ‐ visual stimuli‐ in order to maximize power. Maps were thresholded at a voxel level *p *< 0.01 (*z *> 2.3) and a cluster level at *p *< 0.05, corrected for multiple comparisons. This resulted in five activity clusters (see Table 2): right middle frontal gyrus (rMFG), left frontal eye field (lFEF), left temporoparietal junction (lTPJ), left inferior frontal gyrus (lIFG) and the right superior parietal cortex (rSPC). To localize each brain site bilaterally, we reduced the voxel level threshold to *p *< 0.03 (*z *> 1.8) which allowed us to also determine within our sample the left middle frontal gyrus (lMFG), right frontal eye field (rFEF), right temporoparietal junction (rTPJ) and the right cingulate cortex (rCC). ROIs were then defined by a 5 mm sphere around the peak voxel of each of these nine clusters. Note that some of the ROIs were part of a larger cluster in the whole‐brain analysis. If so, the Harvard Cortical Structure Atlas provided in FSL was used in order to further constrain the peak voxel of the ROI.

**Table 2:** Clusters for ROI definition

Brain region	Cluster size	Max. z	X	Y	Z	z‐threshold
Frontal brain regions						
Left frontal eye field (lFEF)	693	4.1	−42	−2	48	2.3
Right frontal eye field (rFEF)	110	3.16	52	−4	50	1.8
Left middle frontal gyrus (lMFG)	693	3.39	−46	18	38	1.8
Right middle frontal gyrus (rMFG)	887	4.43	42	16	36	2.3
Right cingulate cortex (rCC)	1049	3.1	2	−6	46	1.8
Left inferior frontal gyrus (lIFG)	693	4.1	−58	6	6	2.3
Parietal brain regions						
Right temporo‐parietal junction (rTPJ)	771	2.99	58	−44	28	1.8
Left temporo‐parietal junction (lTPJ)	486	4.15	−60	−48	22	2.3
Right superior parietal cortex (rSPC)	344	3.98	38	−34	46	2.3

*Note*

ROIs were defined based on significant activation in the group comparison (AVGPs > NAVGPs) for the contrast Visual Stimuli versus Baseline. Cluster size refers to voxels in the template brain (1 mm^3^). Coordinates (X,Y,Z) in MNI space refer to the peak voxel with the maximum z‐value.

#### 2.5.3 Psychophysiological interactions

In order to investigate group differences in connectivity, seed‐based psychophysiological interaction (PPI) analyses were performed with FSL using the above defined 9 ROIs as seed regions (O'Reilly, Woolrich, Behrens, Smith, & Johansen‐Berg, 2012). All 9 ROIs have been selected as these brain areas are likely involved in the top‐down attentional control network. A PPI examines whether the correlation of the MR signal time course between a seed region and other brain areas (physiological) is contingent (interacting) on (psychological) contexts such as events of preparation or target processing. For all ROIs, the peak voxel was transformed into each individual's native functional space and then dilated to a spherical region around that voxel by a 5 mm kernel. Then, for each ROI and each participant, a mean fMRI time series was extracted from the preprocessed (i.e., filtered and motion‐corrected) images. Furthermore, the GLM included regressors for cue, SOA and visual stimuli. Next, separate first‐level analyses were performed for each seed at a single‐subject level including a physiological regressor (time course of the seed), the psychological regressor of interest (either cue or visual stimuli in two separate analyses) and the interaction regressor (element‐wise product based on the psychological and physiological regressors). Other events (e.g., SOA) served as regressors of no interest. Additionally, the mean fMRI signal time course in white matter and the cerebrospinal fluid, respectively, were added as nuisance regressors in order to control for physiological noise (Behzadi, Restom, Liau, & Liu, 2007). These were estimated based on a spherical region (3 mm kernel) within each structure. Neither the physiological regressor nor the interaction regressor was convoluted with a hemodynamic response function as they already represented the real‐time state of the brain during scanning. The single‐subject analysis was followed by a between‐group mixed effects analysis using FLAME that compared coefficient maps for the psychophysiological interaction between AVGPs and NVGPs. Again, statistical images were thresholded using clusters determined by voxel level *p *< 0.01 (or *z *> 2.3) and a (corrected) cluster significance threshold of *p *<* *0.05 (Worsley, 2001). We only report significant clusters with peak voxels in gray matter.

#### 2.5.4 Relating brain activation to behavior

In order to examine the relationship between brain activation and behavior, we examined the correlation between the BOLD responses of the selected ROIs and the inverse efficiency scores (target‐present trials) or false alarm rates (catch trials). Before carrying out regression analyses, however, we reduced the dimensionality of the 9 ROIs using a factor analysis. As BOLD signals in the 9 ROIs were partially intercorrelated, the factor analysis (principal component analysis) reduced the BOLD signal variation into a smaller set of independent components. Only components with an Eigen value larger than 1 were selected (Kaiser‐Guttman criterion; Kaiser & Dickman, 1959). Subsequently, a varimax rotation of the reduced component space was performed in order to identify the combination of ROIs that most strongly correlated with the components.

## 3 RESULTS

### 3.1 Behavioral results

#### 3.1.1 Fixation control

Participants had to fixate on a rotating fixation cross in the middle of the screen and count the numbers of missing arms occurring infrequently during the presentation of each block. Overall, participants performed quite well with an average accuracy of more than 93%, indicating that they fixated as instructed most of the time. AVGPs missed 5.76% (SE = 0.61) of the arms of the rotating fixation cross, whereas NVGPs missed 7.83% (SE = 1.40). Although AVGPs had numerically fewer misses, this difference was not significant *t* (30)  = −1.348, *p *=* *0.193), and thus all trials were included in the following analyses for both groups.

#### 3.1.2 Target‐present trials

**Figure 2:**
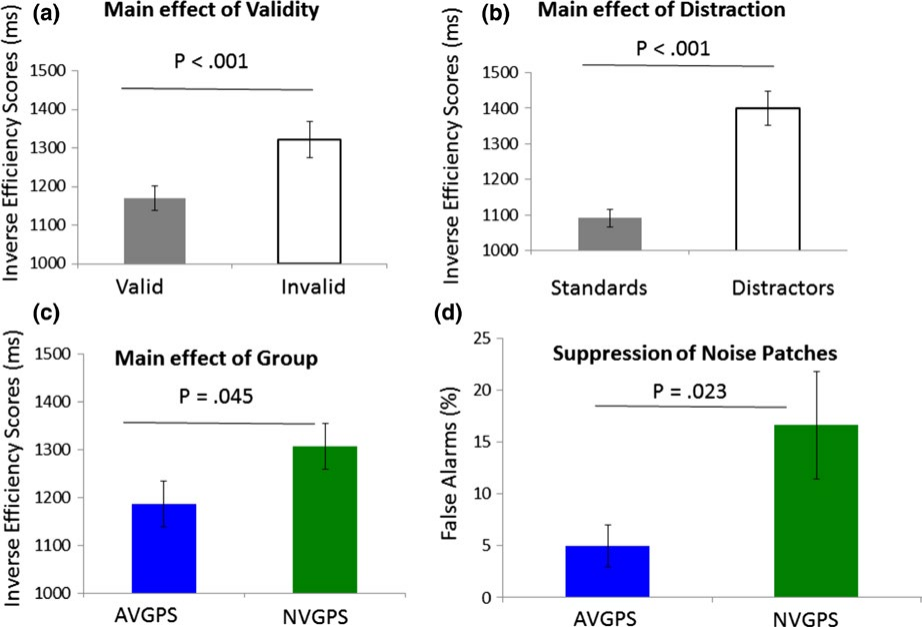
Behavioral results. (a) Inverse efficiency scores for target discrimination were higher for validly cued than for invalidly cued trials. (b) Inverse efficiency scores were higher for standard trial than for distractors trials. (c) Inverse efficiency scores were higher for gamer group (AVGPs) than for the control group (NVGPs). (d) False alarm rates to target‐absent trials were lower in the gamer group (AVGPs) than in the control group (NVGPs). Error bars reflect standard errors of the mean

All analyses were collapsed across both sessions as the factor session interacted with no other factors (see Data S1). Given our interest in group differences, we extracted a single behavioral measure rather than considering RTs and accuracy separately. To this end, we computed an inverse efficiency score defined by reaction times divided by (1‐error rate) (Bruyer & Brysbaert, 2011). This score was calculated separately for each participant and each condition (standard valid, standard invalid, distractor valid, distractor invalid trials). An ANOVA including the factors *Validity* (valid versus invalid trials), *Distraction* (Standards vs. Distractors) and the between‐subject factor *Group* (VGPs vs. NVGPs) on inverse efficiency scores revealed a main effect of *Validity* (*F* (1,30)  = 14.73, *p *<* *0.001; $\eta_{p}^{2}$: 0.329; mean valid: 1170 ms; SE: 31; mean invalid: 1322 ms, SE: 46) and a main effect of *Distraction* (*F* (1,30)  = 90.36, *p *<* *0.001; $\eta_{p}^{2}$: 0.751; mean standards: 1092 ms, SE: 25; mean distractors: 1400 ms; SE: 48). The main effect of *Group* was weak but showed the expected trend of better performance in AVGPs as compared to NVGPs (*F* (1,30)  = 3.04, *p* = 0.045 *one‐tailed*; $\eta_{p}^{2}$: 0.092; mean VGPs: 1186 ms; SE: 48; mean NVGPs: 1306 ms; SE:48). The *Group* factor did not interact with any of the factors (all *ps* > 0.5) (see Figure 2).

#### 3.1.3 Catch Trials

Performance on the catch trials (two noise patches presented instead of a target and a noise patch) was considered separately by looking at false alarm rates as participants were asked to withhold their response on those trials. AVGPs made less false alarms compared to NVGPs (*t*‐test for independent samples: *t* (30)  = 2.09, *p* = 0.023 one‐tailed, mean NVGPs: 16.6%, SE = 5.18, mean AVGPs: 4.98, SE = 2.02) (see Figure 2 d).

### 3.2 Results of the brain imaging analysis

#### 3.2.1 Whole‐brain analyses: within‐group maps and group overlap

**Figure 3:**
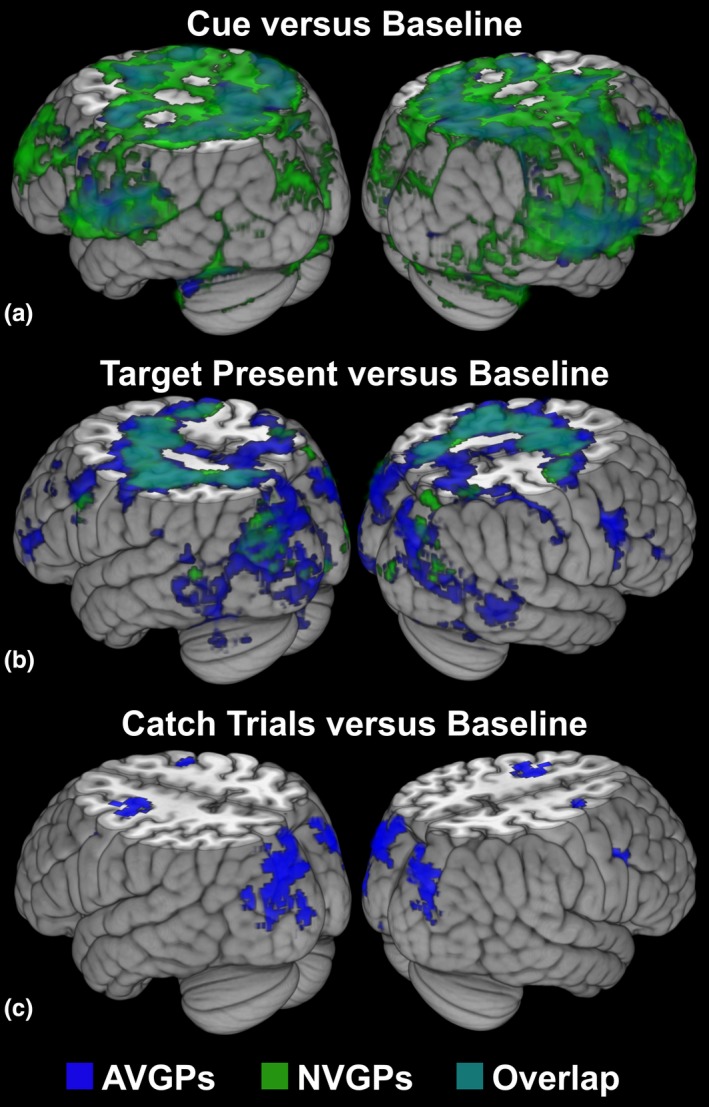
Group maps and their overlap for the contrasts (a). cue period (b). visual stimuli in target‐present trials, and (c). visual stimuli in catch trials. Maps were thresholded using clusters determined by voxel level *p* < 0.01 (or *z *> 2.3) and a (corrected) cluster significance threshold of *p* < 0.05 (Worsley, 2001)

We first considered the network of areas activated in each group and their overlap separately, during the auditory cue period and then during the more bottom‐up target processing period following the presentation of the stimuli (see Figure 3). As described in previous studies (Corbetta & Shulman, 2008 for a review), cue processing recruited in both groups a frontoparietal attentional network including the MFG, FEF and the SPC bilaterally, as well as the cingulum (CC) and the insula. Moreover, during target‐present trials, brain activation patterns specific to reorienting of attention and ignoring distractors, such as the TPJ, were observed (see Corbetta & Shulman, 2008 for a review; Hopfinger et al., 2000; Shulman et al., 2010; Wu et al., 2015), as well as the recruitment of sensory‐specific areas, such as the visual cortex.

The overlap of brain activation confirmed that a similar network of areas was recruited in NVGPS and AVGPs during cue and target‐present periods, albeit to a different extent (see Figure 3). Moreover, similar brain areas were recruited in both groups during the SOA period (see Figure S1). Interestingly, there was little activation in NVGPs and no overlap in brain activation between the two groups for the processing of catch trials (only noise patches). We further examined these differences below by between‐group, whole‐brain, and ROI‐based analyses.

#### 3.2.2 Between‐group whole‐brain analyses

Differences in brain activation across groups were then assessed for the contrasts cue period versus baseline, visual stimuli versus baseline in target‐present trials and noise patches versus baseline in catch trials (see Table 3). Greater activation in the frontoparietal network of attention was observed in NVGPs as compared to AVGPs during the cue period. In contrast, the frontoparietal network of attention was more activated during target or noise patch processing in AVGPs as compared to NVGPs. This difference was especially observed in areas such as frontal pole, the MFG, the postcentral gyrus and the TPJ (see Table 3 for an overview). Note that no group differences were observed during the SOA period, and thus the SOA period was not analyzed further.

**Table 3:** Brain clusters for the group contrasts cue, target‐present and catch trials

Brain region	Cluster size	Max. z	X	Y	Z
CUE <> baseline in NAVGPs > AVGPs					
Right insula	2994	4.25	38	20	−2
Right cerebellum	2103	3.78	28	−62	−24
Right frontal eye field	870	4.08	52	10	36
Left cerebellum	735	3.49	−44	−60	−26
Left occipital	715	4.15	−18	−98	24
Right paracingulate gyrus	710	3.75	6	18	38
Left cingulate gyrus (posterior division)	608	3.62	−6	−24	26
Left cerebellum	403	3.66	−6	−56	−34
Right occipital	375	3.35	24	−90	30
Right supramarginal gyrus (anterior division)	360	3.51	62	−24	32
TARGET−PRESENT <> baseline in AVGPs > NAVGPs					
Left frontal eye field	732	3.92	−46	4	50
Right middle frontal gyrus	648	3.93	40	14	36
Left temporo‐parietal junction	499	3.85	−64	−42	28
CATCH TRIALS <> baseline in AVGPs > NAVGPs					
Left frontal eye field	734	3.75	−40	0	52
Right middle frontal gyrus	724	3.42	40	10	34
Left inferior frontal gyrus	479	3.36	−52	34	16
Left temporo‐parietal junction	422	3.58	−62	−46	20

*Note*

The table only reports main clusters (no subclusters). Cluster size refers to voxels in the template brain (1 mm^3^). Coordinates (X,Y,Z) in MNI space refer to the peak voxel with the maximum z‐value. A cluster‐based thresholding with a voxel level of *p* < 0.01 (*z *> 2.3) and a cluster level of *p* < 0.05 was used.

### 3.3 Between‐group ROI analyses

The 9 ROIs defined above were used to compare group activation within the so‐defined top‐down attentional network for the cue, target‐present and catch trial periods. Three separate ANOVAs were run on the extracted percent signal change in each period, with the within‐subject factor ROI (9 levels) and the between‐subject factor Group (AVGPs vs. NVGPs). For the cue period, reduced percent signal change was observed in AVGPs compared to NVGPs (main effect of *Group*:* F* (1,30)  = 6.98, *p* = 0.013; $\eta_{p}^{2}$: 0.189; mean AVGPs: 0.073, SE = 0.013; mean NVGPs: 0.122, SE = 0.013). The main effect of ROI was significant, indicating regional variations in recruitment strength and in particular, numerically higher percent signal change extracted from the left inferior frontal gyrus (*F* (8,240)  = 13.99, *p *<* *0.001, $\eta_{p}^{2}$: 0.318). The interaction between the factors *ROIs* and *Group* was not significant (*F* (8,240)  = 2.12, *p* = 0.095, $\eta_{p}^{2}$: 0.066), indicating similar regional variations across groups.

For the target‐present period, enhanced percent signal change was observed in AVGPs compared to NVGPs (main effect of *Group*:* F* (1,30)  = 23.58, *p *<* *0.001, $\eta_{p}^{2}$: 0.440; mean AVGPs: 0.050, SE = 0.011; mean NVGPs: −0.024, SE = 0.011). The main effect of ROI was also significant (*F* (8,240)  = 3.203, *p* = 0.027, $\eta_{p}^{2}$: 0.096), indicating regional variations in BOLD activity, and in particular numerically higher percent signal change in the right MFG. The interaction between the factors *ROI* and *Group* was marginally significant (*F* (8,240)  = 2.41, *p* = 0.072, $\eta_{p}^{2}$: 0.074) suggesting overall quite comparable regional variations across groups except for possibly greater AVGPs recruitment in the inferior frontal gyrus.

On catch trials, participants had to reevaluate their task goals and withhold their response as no targets were presented. Higher percent signal change was observed in AVGPs compared to NVGPs (main effect of Group: *F* (1,30)  = 30.71, *p *<* *0.001; $\eta_{p}^{2}$: 0.506; mean AVGPs: 0.008, SE = 0.009; mean NVGPs: −0.061, SE = 0.009). The main effect of ROI was significant, indicating regional variations in recruitment strength and, in particular, with numerically higher percent signal change extracted from the right middle frontal gyrus (rMFG) (*F* (8,240)  = 17.31, *p *<* *0.001, $\eta_{p}^{2}$: 0.366). The interaction between the factors *ROI* and *Group* was not significant (*F* (8,240)  = 1.89, *p* = 0.134, $\eta_{p}^{2}$: 0.059), indicating similar regional variations across groups.

### 3.4 Functional connectivity

#### 3.4.1 Cue period

The PPI analysis time‐locked to the sound cue revealed no group differences between AVGPs and NVGPs in correlated brain activation when seeding from the nine ROIs reported in Table 2.

#### 3.4.2 Visual stimuli period

The PPI analysis time‐locked to visual stimulus presentation (target‐present and catch trial combined—using the same GLM as for defining the ROIs) showed enhanced functional connectivity in AVGPs as compared to NVGPs for seeds in the right FEF, left MFG, right SPC and left IFG (see Table 4). Interestingly, areas of the top‐down attentional control network showed both enhanced functional connectivity with sensory related areas and with frontoparietal areas of attentional control. For instance, the activity in seeds of the right FEF showed greater connectivity with sensory areas such as the central and parietal operculum – brain areas reported to be involved in auditory and somatosensory processing (Pleger et al., 2003; Elmer, Meyer, Marrama, & Jäncke, 2011). Similarly, enhanced connectivity in AVGPs was observed in the lateral occipital cortex and the intracalcarine sulcus for a seed in the right SPC. Moreover, activity of seeds in the left MFG of AVGPs exhibited greater connectivity with frontoparietal areas such as the left paracingulate gyrus and the left central opercular gyrus, extending to the parietal opercular gyrus and the postcentral gyrus.

**Table 4:** Functional connectivity for the visual stimuli period

Seed region	Brain region	Cluster size	Max. z	X	Y	Z	COG X	COG Y	COG Z
Left FEF	–	–	–	–	–	–	–	–	–
Right FEF	Left central opercular cortex	887	3.8	−46	−20	20	−40	−18	26
	Left parietal opercular cortex		3.42	−42	−24	24			
	Left parietal opercular cortex		3.39	−36	−26	20			
	Left precentral gyrus		3.23	−46	−10	34			
Left MFG	Left paracingulate gyrus	904	3.27	−6	−40	22	−4	26	28
	Left paracingulate gyrus		3.09	−6	46	22			
	Left paracingulate gyrus		3.08	−10	20	32			
	Left paracingulate gyrus		3.0	−4	36	32			
	Left supplementary motor area (juxtapositional lobule cortex)		2.93	−10	4	42			
	Left paracingulate gyrus		2.87	−8	34	24			
Right MFG	–	–	–	–	–	–	–	–	–
Left TPJ	–	–	–	–	–	–	–	–	–
Right TPJ	–	–	–	–	–	–	–	–	–
Right CC	–	–	–	–	–	–	–	–	–
Right SPC	Left lateral occipital cortex	3019	3.66	−40	−64	10	−40	−64	10
	Left middle temporal gyrus		3.56	−48	−62	10			
	Right intracalcarine cortex		3.4	12	−80	10			
	Right intracalcarine cortex		3.2	6	−84	0			
	Left precuneus cortex		3.2	−4	−60	10			
	Left lateral occipital cortex		3.19	−40	−88	22			
Left IFG	Left postcentral gyrus	481	3.63	−50	−24	34	−46	−24	38
	Left postcentral gyrus		3.07	−42	−24	46			
	Left postcentral gyrus		2.94	−48	−22	46			
	Left postcentral gyrus		2.91	−46	−26	54			
	Left precentral gyrus		2.89	−46	−12	34			
	Left postcentral gyrus		2.67	−42	−24	58			

*Note*

Cluster locations and z‐values of significant group differences in the contrast visual stimuli versus baseline. Labels of brain region refer to the peak voxel x y z coordinate. Subclusters are reported as well. COG = center of gravity. Note that enhanced connectivity was only observed for the AVGP group (AVGP > NAVGP), but not for the NAVGP group (NAVGP > AVGP). A cluster‐based thresholding with a voxel level of *p* < 0.01 (*z *> 2.3) and a cluster level of *p* < 0.05 was used.

### 3.5 Relating brain activation to behavior

**Figure 4:**
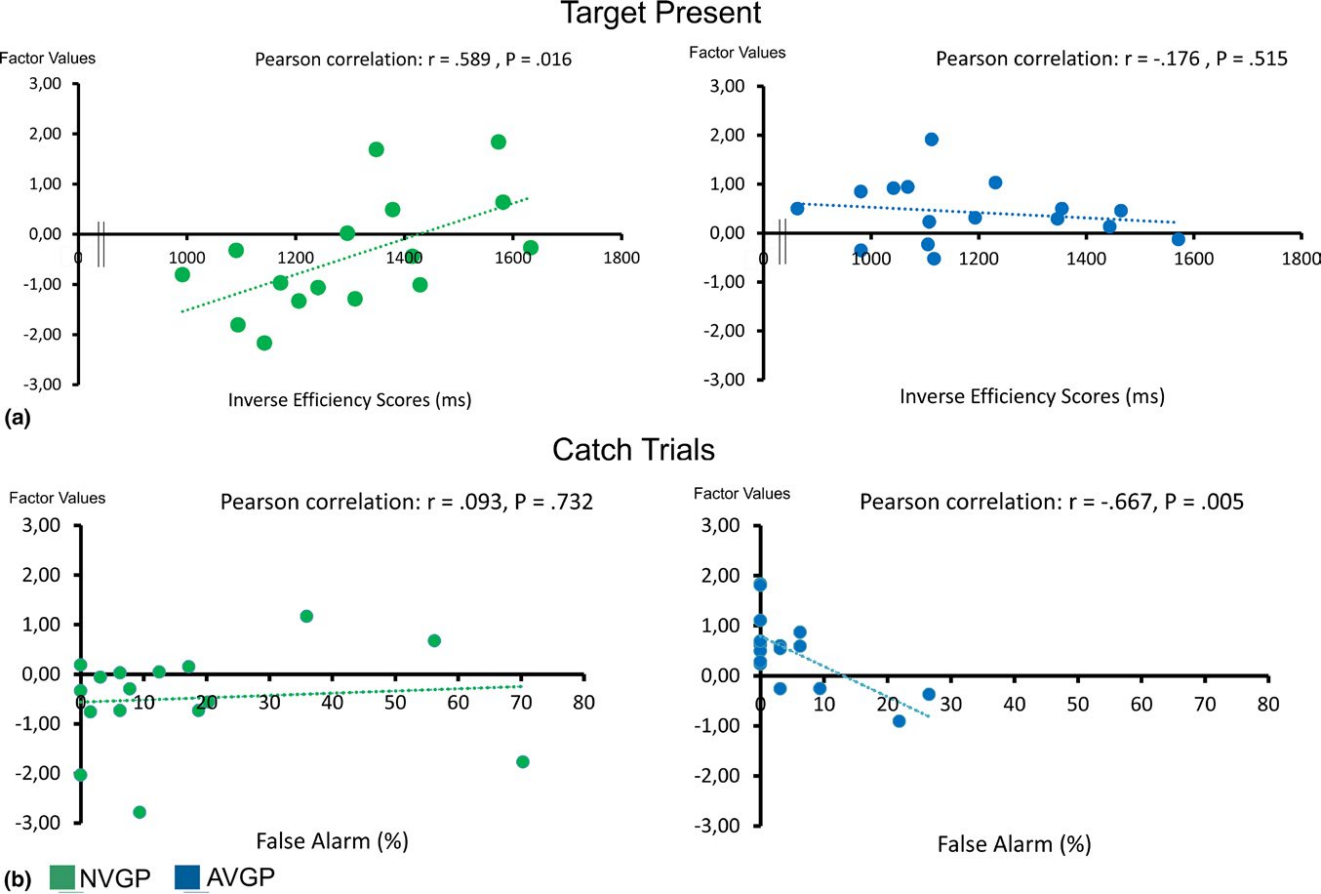
Correlations between extracted percent signal change (rotated factor scores) and behavioral performance in AVGPs (blue) and NVGPs (green). (a). Correlation between factor values of the 2nd component for target‐present trials and inverse efficiency scores. (b) Correlation between factor values of the 1st component for catch trials and false alarm rates

The factor analysis on target‐present activation in the 9 ROIs identified two main components. After varimax rotation, the first (primary) component loaded most strongly (explained variance >50%) on the right TPJ, right MFG and right SPC. The second component loaded most strongly on the right FEF and the left MFG (see Table S1). We then ran a multiple regression analysis based on the factor values of these two components. The second component was found to correlate significantly with the inverse efficiency scores in the NVGPs group (Figure 4a). Greater activation along this second component (right FEF and left MFG) in NVGPs was linked to higher inverse efficiency scores or, in other words, poorer performance (beta = 0.589, T = 2.72, *p* = 0.016).

The factor analysis on catch trial activation also revealed two components. The first component loaded most strongly on the right TPJ, right MFG, and right SPC. The second component loaded most strongly on the left TPJ, the left MFG, and the right and left FEF (see Table S1). A subsequent regression analysis indicated that the first component (right TPJ, right MFG, and right SPC) correlated significantly with false alarm rates in AVGPs (Figure 4b). Greater activation along this first component corresponded to less false alarms, or better performance, in AVGPs (beta = −0.667, *T* = −3.35, *p* = 0.005).

## 4 DISCUSSION

The major aim of the present study was to investigate how the brain networks mediating attentional control are recruited in the face of more efficient attentional control. For this purpose, a cross‐modal Posner‐cueing paradigm was used in which participants were directed via an auditory cue to the most likely location of an upcoming Gabor patch target and asked to discriminate the orientation of the Gabor patch target as fast and accurately as possible. In 40% of the trials, participants had to either reorient their attention after an invalid cue, ignore distractors or withhold their response, ensuring sufficient variations in attentional demands to elicit tight attentional control.

As expected, all participants showed enhanced performance in validly cued trials as compared to invalidly cued trials and responded more efficiently to stimuli presented without as compared to with high contrast flanking distractors. In addition, AVGPs were better able to suppress responses in catch trials during which only noise patches were presented. They also showed the expected trend for better performance in target‐present trials, as measured by the ratio of RTs to accuracy rate, as compared to NVGPs.

The neuroimaging data revealed several group differences concerning the frontoparietal network of attentional control under study. We found a marked reduction in AVGPs activation upon hearing the auditory cue and thus when participants had to initially orient their attention. By contrast, during visual processing (both, target‐present and catch trials), higher activation was observed in AVGPs as compared to NVGPs. Although this group difference was observed across several brain areas, it was most pronounced in the temporal parietal junction (TPJ) and the middle frontal gyrus (MFG). Besides those more general effects during visual processing, there were also specific group differences in target‐present and in catch trials. The extracted percent signal change during target‐present trials was related to task performance in NVGPs only with a higher activation in the right FEF and left MFG being linked to higher IE scores, or in other words worse performance. Such greater activation may therefore be interpreted as a sign of more effortful target processing. In contrast, during catch trials extracted percent signal change in the right MFG, right TPJ and right superior parietal cortex was correlated with less false alarms in AVGPs. Thus, greater recruitment of these areas may therefore index better inhibitory control in AVGPs upon viewing the noise patches of catch trials.

Greater overall activation in AVGPs during the processing of visual stimuli was complemented by enhanced functional connectivity as compared to NVGP during that same time‐period from four out of the nine ROIs (see Table 4). For instance, seeding from areas of the frontoparietal network, such as the right FEF and the right superior parietal cortex led to higher functional connectivity in AVGPs as compared to NVGPs toward sensory areas, including visual areas but also areas related to auditory processes. In addition, seeding from the left MFG revealed higher correlated activity in AVGPs and reduced functional connectivity in NVGPs in the paracingulate gyrus, also involved in executive control (Wu, Weissman, Roberts, & Woldorff, 2007), suggesting greater cross‐talk within the frontoparietal network of attentional control in AVGPs.

### 4.1 Higher automatization in AVGPs during task orientation

Upon presentation of the auditory cue, AVGPs showed reduced frontoparietal network recruitment as compared to NVGPs. This difference can be interpreted in various ways, as reduced activation could be linked to either *zooming out of the current task* or processing being *more automatic*. For instance, AVGPs may have zoomed out and ignored the auditory cue during the SOA period before the target is presented, and rather only boosted their attention when target processing is relevant. Alternatively, AVGPs may benefit from more automatic attentional control than NVGPs by which they may consistently focus their attention during a sustained time‐period but in an effortless manner. Several aspects of the findings argue in favor of the latter hypothesis. First, even though activation was reduced in AVGPs as compared to NVGPs, AVGPs still displayed the expected recruitment of the frontoparietal attentional control network during the cue and waiting periods (see Figure 3). Second, at the behavioral level, AVGPs exhibited a robust validity effect indicating that they have reoriented their attention according to the auditory cue, and thus have not zoomed out of the current task demands. Finally, during the target period, AVGPs were better able to withhold their response when noise patches are presented, which confirms they are “on‐task” during the experiment.

The proposal that the reduced attentional network recruitment in AVGPs during cue processing may be a neural signature of efficient attention allocation is in line with both previous behavioral studies documenting enhanced attentional control in AVGPs and brain imaging studies documenting decreased activation with automatization. Several behavioral studies have documented that AVGPs benefit from more efficient attentional control (Green & Bavelier, 2012 for a review). AVGPs showed enhanced distributed attention (Belchior et al., 2013; Dye & Bavelier, 2010; Feng, Spence, & Pratt, 2007; Green & Bavelier, 2003, 2006; Wu & Spence, 2013) and are able to track multiple moving objects more swiftly than NVGPs, indicating higher abilities for maintaining attention over a sustained period of time (Dye & Bavelier, 2010; Green & Bavelier, 2006; Trick, Jaspers‐Fayer, & Sethi, 2005). They also showed reduced attentional blink and increased change detection (Cain, Prinzmetal, Shimamura, & Landau, 2014; Clark et al., 2011), and they have been reported to better suppress irrelevant information (Chisholm & Kingstone, 2012; Chisholm et al., 2010; Mishra et al., 2011). Together, the available studies converge in showing enhanced attentional control in AVGPs.

The view that AVGPs may benefit from a greater automatization in attention allocation is also in line with the literature which has repeatedly documented reduced brain activation as automatization sets in. For instance, Armbuster and coauthors have documented a reduced activation and functional coupling in frontal brain areas in individuals with high cognitive flexibility (Armbruster et al., 2012), whereas others have observed that repeated practice leads to reduced brain activation along with performance improvements (Asaad et al., 1998; Beauchamp et al., 2003; Erickson et al., 2007; Jansma et al., 2001; Landau et al., 2004; Milham et al., 2003; Raichle et al., 1994; Schneiders et al., 2012). Although further confirmation is needed, a plausible explanation for the link between automatization and lesser activation is that the computations required to accomplish the same processing demands fewer resources in AVGPs, which might be linked to a more efficient integration of information in the service of decision making (Bavelier et al., 2012; Green, Pouget, & Bavelier, 2010).

### 4.2 Target processing efficiency and the TPJ

When participants were asked to identify the orientation of the target stimulus, higher activation was observed in AVGPs compared to NVGPs for both, target‐present and catch trials. One major region of interest in which we observed group differences is the TPJ, which generally refers to an area of cortex at the junction of the inferior parietal lobule, lateral occipital cortex, and the posterior superior temporal sulcus (see Carter & Huettel, 2013 for a review; Corbetta et al., 2008; Donaldson, Rinehart, & Enticott, 2015; Geng & Vossel, 2013). The TPJ receives inputs from thalamic, limbic, somatosensory, visual and auditory areas and has bidirectional connectivity with distal temporal and prefrontal regions (Carter & Huettel, 2013; Corbetta et al., 2008; Donaldson et al., 2015). Although there is general agreement that the TPJ is strategically located to regulate the interplay between top‐down and bottom‐up attention, the mechanisms that are mediated by the TPJ are still debated. According to the model proposed by Corbetta & Shulman (2002) for a review, the TPJ acts as a “circuit breaker” which sends inhibitory signals to frontal and parietal areas of the top‐down network, in order to summon attention to a new, task‐relevant stimulus. The present results are in line with a more efficient “circuit‐breaking” system in AVGPs compared to NVGPs, as indexed by the link between greater TPJ activation and better performance in AVGPs during catch trials. Indeed, while participants are expecting to select a response, this behavioral pattern needs to be suppressed during catch trials as no target is presented.

Note that the “circuit‐breaking” function of the TPJ has been recently questioned by some authors (Geng & Vossel, 2013). If the TPJ has the function of a “circuit breaker”, one might expect the TPJ to send *earlier* signals to the frontal areas than vice versa. TMS and EEG studies suggest that the TPJ might come online at a later time than the frontal areas. Indeed, TMS effects over the TPJ are reported 150–250 ms after stimulus onset (Meister et al., 2006) while TMS impact over frontal areas (such as the right FEF) is visible as early as 40 and 80 ms after stimulus onset (see Geng & Vossel, 2013 for a review; O'Shea, Muggleton, Cowey, & Walsh, 2004). Additionally, the P300, an event‐related potential observed about 300 ms after stimulus onset and linked to stimulus evaluation on the way to elaborating a decision, has been linked to the TPJ. Interestingly, a major function ascribed to the P300 is “contextual updating” or the mechanisms by which participants change their internal model of the environment in response to external stimuli (Donchin & Coles, 1988; Polich, 2007). In this view, greater TPJ recruitment in AVGPs might reflect their greater flexibility in adapting their internal models to environmental changes. Enhanced amplitudes in the P300 have been shown in AVGPs compared to NVGPs in a few studies before (Mishra et al., 2011; Wu et al., 2012) which have been interpreted so far as being in line with the more efficient decision making in AVGPs documented in other studies (Green et al., 2010).

A third view assigns a “filter” role to the TPJ. For instance, when participants are asked to detect a target among distractors, the TPJ deactivates until the target is detected. Shulman et al. (2007) have suggested that the deactivation reflects the filtering of irrelevant inputs from the TPJ and preventing unimportant objects from being attended. In a fraction of trials, high contrast Gabor patches were additionally presented between the fixation cross and target Gabors, and had to be ignored to successfully perform the task. However, AVGPs did not show additional improved distractor suppression performance compared to NVGPs but exhibited only a main effect of Group across all conditions. Therefore, that latter interpretation of the TPJ function to explain our group difference seems less likely. In sum, the AVGP advantage during catch trials appears most consistent with a swifter contextual updating or more agile circuit breaker view of the TPJ in this population.

### 4.3 Regulating the interaction between top‐down and bottom‐up attentional control

Interestingly, various imaging techniques have provided evidence for the involvement of the parietal cortex in both top‐down and bottom‐up orienting (Corbetta & Shulman, 2002; Corbetta et al., 2008; Uncapher, Hutchinson, & Wagner, 2011). For instance, areas along the dorsal parts of the parietal cortex, such as the superior parietal lobule (SPL) and the inferior parietal lobule (IPL), are activated following top‐down cues prompting participants to attend to particular locations, whereas ventral areas of the parietal cortex such as the TPJ, are more involved in bottom‐up related attentional orienting.

The neural mechanisms behind the more flexible updating of task conditions in AVGPs, as exemplified by their lesser false alarm rate in catch trials, might be due to enhanced correlated activity across bottom‐up and top‐down‐related areas. Accordingly, seeding from the right superior parietal cortex (an area of the top‐down attention network), showed enhanced correlated activation in occipital areas in AVGPs compared to NVGPs.

This finding is in line with previous animal and human studies demonstrating enhanced coupling between frontoparietal and visual brain areas in fMRI (Büchel & Friston, 1997; Buschman & Miller, 2007; Gilbert & Li, 2013) and MEG studies (for example Siegel, Donner, Oostenveld, Fries, & Engel, 2008). The fact that AVGPs exhibited improved behavioral performance when confronted with noise patches might suggest that the top‐down connections (parietal‐visual) are more efficiently used in AVGPs compared to NVGPs during visual stimulus processing. Thus, enhanced connectivity in AVGPs compared to NVGPs is in line with the observation that the voluntary allocation of visuospatial attention depends upon top‐down influences from the FEF and intraparietal sulcus (IPS)—the core regions of the dorsal attention network (DAN)—to visual occipital cortex (VOC) (Meehan et al., 2017) and other brain areas involved in sensory processing.

Uncapher and Wagner (2009) have suggested the dual attention encoding hypothesis, which states that top‐down and bottom‐up attention may differentially foster encoding success and failure. The assumption is that top‐down attention enhances cortical representations for attended information. In contrast, engagement of the ventral parietal cortex may lead to later memory failure when attention is captured by irrelevant information. Following this hypothesis, it might be argued that top‐down attention in AVGPs enhances the cortical representation of information to a higher extent than in NVGPs. On the other hand, AVGPs may be more resistant to irrelevant information, as suggested by increased suppression of noise patches and higher activation in the TPJ.

Besides enhanced connectivity to visual areas when seeding from the superior right parietal cortex, we observed enhanced correlated activity in the paracingulate gyrus, when seeding from the left MFG, two brain areas that belong to the top‐down attentional control network. The cingulate cortex has been reported to be involved in higher attentional control functions (Aarts & Roelofs, 2011) but in particular the regulation of possible conflicts between top‐down task goals and bottom‐up stimulus.

The present results are in line with recent studies. Daily gaming performance has been associated with improved working memory performance in adolescents as well as increased recruitment of the frontoparietal network during working memory (n‐back) tasks (Moisala et al., 2016). An enhanced integration of top‐down and bottom‐up networks has also been recently documented in individuals who play Multiplayer Online Battle Arena (MOBA) games. Interestingly, this gaming difference seems to be related to the integration between a sensory bottom‐up network and a central executive, top‐down network, as documented by tighter correlations during resting state analyses (Gong et al., 2016). Although these effects along with those of the present study point into similar changes, more work is needed to characterize the impact of video game playing and its many genres on the top‐down and bottom‐up attentional networks and their interaction.

## 5 SUMMARY AND CONCLUSION

We contrasted AVGPs and NVGPs in a Posner‐cueing paradigm to characterize how the attentional control network is altered in AVGPs, a population which benefits from greater attentional control. Strikingly, whether greater or lower recruitment was a marker of better attentional control depended on processing requirements. During the cue period, reduced recruitment of the frontoparietal network characterized greater attentional control, especially in frontal regions. In contrast, during the target period, enhanced recruitment characterized greater task efficiency as measured for example by lesser false alarms, especially in the right MFG, the right TPJ and the right SPC. Interestingly, during the processing of visual stimuli, the connectivity between top‐down brain areas and perceptual areas was strengthened in AVGPs, maybe a signature of higher attentional control. The interaction between top‐down and sensory areas appeared mainly regulated by the right TPJ and the right MFG, two key areas in mediating more efficient attentional control mechanisms.

## CONFLICT OF INTEREST

None of the authors have any conflicts of interest associated with this study.
