# Supplementary material for: Neural bases of enhanced attentional control: Lessons from action video game players
Source: Brain Behav. 2018 Jun 19;8(7):e01019. doi: 10.1002/brb3.1019 (PMC6043695; doi:10.1002/brb3.1019)
Supplement: Supplementary file 1 [file BRB3-8-e01019-s001.docx]

**Supplementary Material**

S1. Behavioral Results separate for Session 1 and Session 2

S2. Results of functional imaging analysis for contrast SOA versus Baseline (text, Figure S1)

S3. Results of factor analyses (Table S1)

**S1. Behavioral Results separate for Session 1 and Session 2**

In this analysis, we included only those participants, who have participated in session 1 and in session 2, that is 16 AVGPs and 15 NVGPs. Within-subject factors of interest included Sessions (session 1 or session 2), Validity (valid versus invalid trials), Distraction (Standards versus Distractors), and the between subject factor of interest was Group (AVGPs versus NVGPs). We first ran separate omnibus ANOVAs on reaction times and on error rates for those trials including a target (all but catch-trials). The analyses were based on all participants who took part in session 1 and in session 2 (N = 16 AVGPs, N = 15 NVGPs as one NVGP did not participate in session 2). Reaction times were calculated on correct trials only. Reaction times faster than 200 ms, and slower than 3 seconds were discarded from the analyses.

These analysis confirmed faster RTs in session 2 than session 1 (*F*(1,28) = 44.588, *P* < .001, *η_p_^2^*: .614; session 1: mean: 1163, SE = 27; session 2: mean: 1039, SE = 31) as expected since, in session 2 , stimulus contrast was higher and participants were already familiar with the task. Importantly, the session factor did not interact with any other factors.

Faster RTs and less errors were observed for validly cued as compared to invalid cued trials (*F*(1,28) = 22.704, *P* < .001, η*_p_^2^*: .448 for RTs and *F*(1,29) = 5.131, *P* = .031, *η_p_^2^*: .150 for errors) replicating well known cueing effects, and for stimulus type (*F*(1,28) = 47,621, *P* < .001, *η_p_^2^*: .630 for RTs , *F*(1,29) = 44.553, *P* < .001, *η_p_^2^* .606, for errors) indicating as expected that trials with distractors were harder than those without. Finally, both RTs and error analyses indicated an interaction between validity and distraction due to larger differences in the distractor trials compared to standard trials between valid and invalid conditions, for both reaction times and errors (reaction times: *F*(1,28) = 3.635, *P* = .067, *η_p_^2^*: .115; errors: *F*(1,29) = 5.288, *P* = .029, *η_p_^2^*: .154).

**S2. Results of functional imaging analysis for the contrast SOA versus Baseline**

During the waiting period (SOA period), a similar network of brain areas was recruited in AVGPs and NVGPs. These include bilateral activation patterns in the frontal eye field (FEF), the medial frontal gyrus (MFG), the parietal areas. There were no major differences in brain network recruitment in AVGPs compared to NVGPs (see Figure S1). Between-group analyses did not show any significant group differences in AVGPs compared to NVGPs and in NVGPs compared to AVGPs with a z > 2.3.

Even though the whole brain analyses did not provide any significant group differences in AVGPs compared to NVGPs, we extracted percent signal change from the target regions (defined in table 1). These results again showed, that there are no major differences in AVGPs compared to NVGPs. The ANOVA including the factors ROIs (9 ROIs) and Group (AVGPs versus NVGPs) revealed a main effect of ROI (*F*(8,240) = 10.614, *P* < .001, *η_p_^2^*: .261. The main effect of Group was not significant (*F*(1,30) = .478, *P* = .494, *η_p_^2^*: .016. Moreover, the interaction between ROI and Group was not significant (*F*(8,240) = 1.014, *P* = .383, *η_p_^2^*: .033.


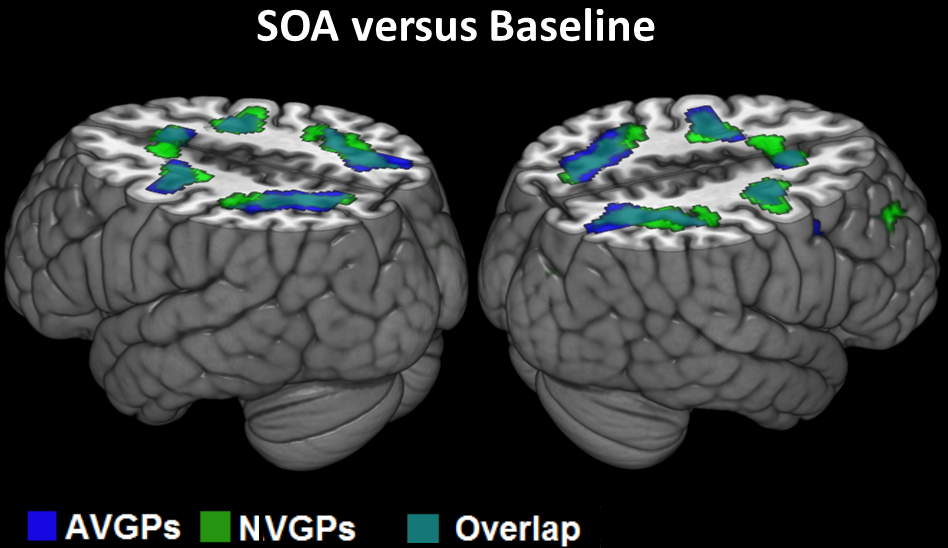


**Figure S1.** Group maps and their overlap for the contrast SOA period.

Statistical maps for the SOA period, in each participant group.

Maps were thresholded using clusters determined by voxel level p < 0.01 (or z > 2.3) and a (corrected) cluster significance threshold of p < .05 (Worsley et al., 2001).

**S3. Results of factor analyses**

**Table S1.** Results of the factor analyses.

| ROI | 1st component | 2nd component |
| --- | --- | --- |
| *TARGET PRESENT <> baseline* | | |
| right TPJ | **.890** | -.206 |
| left TPJ | .529 | .483 |
| right MFG | **.818** | .343 |
| left MFG | .232 | **.729** |
| right FEF | .043 | **.908** |
| left FEF | .653 | .435 |
| right SPC | **.693** | .513 |
| left IFG | .543 | .447 |
| right CC | .584 | .537 |
| *CATCH TRIALS <> baseline* | | |
| right TPJ | **.888** | .031 |
| left TPJ | .464 | **.712** |
| right MFG | **.752** | .229 |
| left MFG | -.116 | **.849** |
| right FEF | .283 | **.739** |
| left FEF | .360 | **.756** |
| right SPC | **.758** | .334 |
| left IFG | .647 | .178 |
| right CC | .377 | .403 |

Note: The table shows the factor loadings for the two selected components (eigenvalue > 1) of the principal component analysis after varimax rotation. For each ROI with explained variance by the component exceeding 50% (square of factor loading > .5) are highlighted in bold. Top refers to the BOLD signal for the contrast Target vs. baseline in target-present trials. Bottom refers to the BOLD signal for the noise patches vs. baseline in catch trials.
